# Sleep and Pain: A Systematic Review of Studies of Mediation

**DOI:** 10.1097/AJP.0000000000000697

**Published:** 2019-03-01

**Authors:** Daniel Whibley, Nourah AlKandari, Kaja Kristensen, Max Barnish, Magdalena Rzewuska, Katie L. Druce, Nicole K.Y. Tang

**Affiliations:** * Epidemiology Group, School of Medicine, Medical Sciences and Nutrition; † Aberdeen Centre for Arthritis and Musculoskeletal Health; # Health Services Research Unit, University of Aberdeen, Aberdeen; ¶ Evidence Synthesis and Modelling for Health Improvement (ESMI), Institute for Health Research, College of Medicine and Health, University of Exeter, Exeter; †† Department of Psychology, University of Warwick, Coventry; ** Arthritis Research UK Centre for Epidemiology, Institute of Inflammation and Repair, University of Manchester, Manchester Academic Health Science Centre, Manchester, UK; ‡ Department of Physical Medicine and Rehabilitation, Kratz Lab; § Department of Anesthesiology, Chronic Pain and Fatigue Research Center, University of Michigan, Ann Arbor, Michigan; ∥ Faculty of Human and Health Sciences, University of Bremen, Bremen, Germany

**Keywords:** systematic review, mediation analysis, pain, sleep problems

## Abstract

**Objectives:**

A relationship between sleep and pain is well established. A better understanding of the mechanisms that link sleep and pain intensity is urgently needed to optimize pain management interventions. The objective of this systematic review was to identify, synthesize, and critically appraise studies that have investigated putative mediators on the path between sleep and pain intensity.

**Methods:**

A systematic search of 5 electronic bibliographic databases (EMBASE, MEDLINE, CINAHL, PsycINFO, and the Cochrane Central Register of Controlled Trials) was conducted. Eligible studies had to apply a formal test of mediation to variables on the path between a sleep variable and pain intensity or vice versa. All searches, data extraction and quality assessment were conducted by at least 2 independent reviewers.

**Results:**

The search yielded 2839 unique articles, 9 of which were eligible. Of 13 mediation analyses, 11 investigated pathways from a sleep variable to pain intensity. Putative mediators included affect/mood, depression and/or anxiety, attention to pain, pain helplessness, stress, fatigue, and physical activity. Two analyses investigated pathways from pain intensity to a sleep variable, examining the potentially mediating role of depressive symptoms and mood. Although evidence supported a mediating role for psychological and physiological aspects of emotional experiences and attentional processes, methodological limitations were common, including use of cross-sectional data and minimal adjustment for potential confounders.

**Discussion:**

A growing body of research is applying mediation analysis to elucidate mechanistic pathways between sleep and pain intensity. Currently sparse evidence would be illuminated by more intensively collected longitudinal data and improvements in analysis.

## Abstract 2

Supplemental Digital Content is available in the text.

The majority of people who live with chronic pain experience poor quality sleep^1^,^2^ and it has been estimated that those with chronic pain are 18 times more likely than their pain-free counterparts to meet the criteria for a clinical diagnosis of insomnia.^3^ The relationship between sleep and pain has been summarized in a number of systematic reviews of both correlational and experimental studies.^2^,^4^,^5^ Although a bidirectional relationship has been observed between these troubling symptoms, compelling evidence suggests that poor sleep is a greater driver of worse pain rather than vice versa.^2^ This interpretation is supported by findings from prospective studies with longitudinal or microlongitudinal (intensive data collection) designs that have recruited adults and children with a range of painful conditions,^6^–^10^ as well as in a general population sample^11^ and in the context of a randomized clinical trial.^12^ The temporal precedence of sleep in the relationship can be conceptualized through a biopsychosocial framework, with likely interconnected mechanisms incorporating the central and autonomic nervous systems, inflammatory responses, cognitions, mood, and behaviors.^2^,^13^–^18^

This direction of inferred causality has important clinical implications; if improvements in sleep lead to reductions in pain, then sleep, as a potentially modifiable behavior, may be a viable target for interventions that aim to reduce pain intensity. However, a meta-analysis of the effect of nonpharmacological interventions to improve sleep has demonstrated modest effects on pain intensity.^19^ Developing an understanding of the mechanisms by which improvements in sleep may lead to improvements in pain may assist in informing and optimizing the content of complex, hybrid interventions for chronic pain that include a sleep improvement component.^20^

Mediation analysis can be used to investigate the relative importance of factors that may lie on the path between an exposure and an outcome. Mediation analysis has its origin in the causal steps approach, where the association between an exposure and an outcome is compared before and after conditioning on the possible mediator.^21^ Methodological advances have led to more sophisticated techniques to determine the existence, magnitude, and statistical significance of “mediated” effects. These techniques, underpinned by the counterfactual framework, partition the total effect of an exposure on an outcome into direct (ie, exposure to outcome) and indirect effects (ie, exposure to outcome “mediated” through an intermediary variable).^22^,^23^ Whether or not the effect of an exposure on an outcome is affected wholly (referred to as total mediation) or partly through the mediator (referred to as partial mediation) can then be assessed, with the statistical significance of any effects determined using specialized tests, for example, a Sobel test^24^ or bootstrapped confidence intervals. Techniques for mediation analysis continue to be improved and refined. Alongside these developments, access to software that allows execution of mediation analysis has enabled researchers to attempt to unravel possible causal pathways pertinent to a range of healthcare-related relationships. Indeed, this has been identified as an important area for further research in the sleep and pain field.^2^,^5^

Despite the relative ease of conducting mediation analysis, its use, particularly when applied to observational data, has been subject to (well-intentioned and well-placed) criticism.^25^ Particular concerns that may render results questionable include: (1) the use of cross-sectional data; (2) imprecision of measurement (particularly when variables are self-reported); and (3) the unlikeliness that confounders will be fully accounted for. Although these concerns may also be levelled at any studies that use observational data, they are particularly pertinent when the research question concerns how change in an exposure may lead to a change in a putative mediator, and how changes in the putative mediator may then influence a change in the outcome. The first of these concerns may be alleviated by the use of prospectively collected data, ideally within the context of a randomised trial, where the exposure can be manipulated, and/or studies with comprehensive and intensive longitudinal data collection (eg, ecological momentary assessment). In the sleep-pain field, the second issue—that of measurement error—is particularly salient. Sleep and pain are multifaceted human experiences, different facets of which can be quantified either subjectively or objectively. When investigating the relationship between sleep and pain it is crucial to be specific about the facet under scrutiny; different facets are not synonymous and may be related to each other through other mediating variables.

A systematic synthesis of studies that have applied formal tests of mediation to investigate variables on the path between sleep and pain intensity is lacking. The tests described in such studies provide estimates of the magnitude of the effect of a sleep variable on pain intensity (or vice versa) that is transmitted through a putative mediating variable (see Fig. 1 for the prototypical case of a single mediating variable). Bringing together and appraising research conducted in this area would help to identify: hypothesized causal pathways that have been investigated, key areas for continued research focus (as well as areas yet to be tapped), and aspects of study design and analysis that may require particular consideration to ensure high quality results. The aim of this systematic review was, therefore, to identify, synthesize and critically appraise studies that have investigated potentially mediating variables on the pathway between sleep variables and pain intensity using a formal test of mediation. Specifically, we: (1) highlight putative mediators that have been investigated and assess the quality of the current evidence; (2) highlight what is missing from the broader picture of investigations into mediators on the path between sleep variables and pain intensity; and (3) make methodological recommendations for future studies.

**FIGURE 1:**
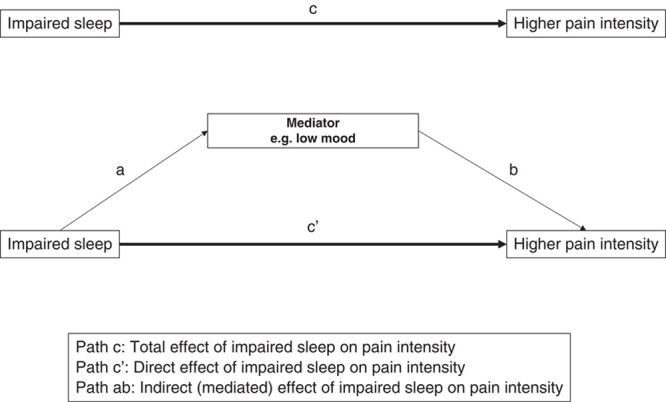
Prototypical case of a single mediating variable on the path from impaired sleep to higher pain intensity.

## MATERIAL AND METHODS

This systematic review was conducted in accordance with the Centre for Reviews and Dissemination’s guidance for undertaking reviews in healthcare^26^ and reported adhering to PRISMA guidelines.^27^

### Search Strategy

Five databases were searched on March 8, 2018 with no start date restriction (EMBASE, MEDLINE, CINAHL, PsycINFO and the Cochrane Central Register of Controlled Trials). The search strategy combined 3 sets with an “AND” Boolean operator: “sleep,” “pain,” and a set previously developed and adapted to detect studies of formal tests of mediation in both observational and experimental studies.^28^ The complete search strategy is presented in Supplement 1 (Supplemental Digital Content 1, http://links.lww.com/CJP/A563). Reference lists of all eligible articles were checked to ascertain whether studies that were not detected by the search strategy could be identified. Key words from eligible articles were also used to search Google Scholar to identify any other eligible studies.

### Eligibility Criteria

Eligible articles were:

1. observational studies (cross-sectional or longitudinal) or randomised controlled trials with
2. a measure of sleep and
3. a measure of pain intensity and
4. a measure of a putative mediating variable with
5. a formal test of mediation (eg, causal steps approach, product of coefficient approach) or a test of the significance of mediated effects (eg, Sobel test or bootstrapped confidence intervals)
6. published in full in a peer-reviewed journal.

We made no content-related restrictions with regard to what may or may not be a reasonable mediator of the sleep-pain or pain-sleep relationship, and included any variable defined as a “mediator” as per the specific criteria of the formal tests of mediation that the primary studies used. There were no time or language restrictions.

### Selection Processes

After conducting database searches and exporting results, duplicate references were removed. Two independent reviewers (N.A. and K.K.) then screened titles and abstracts. Complete texts of articles that met eligibility criteria, or articles where it was not possible to judge eligibility from the title or abstract, were retrieved for further assessment. Two independent reviewers (N.A. and K.K.) evaluated all full-text articles against eligibility criteria. Any differences of opinion or uncertainty regarding eligibility were discussed with a third reviewer (D.W.) until consensus was reached.

### Data Extraction

For each included study, a pair of reviewers independently extracted data (N.A. and K.K.; K.L.D. and M.B.; M.R. and D.W.), with any disagreements discussed and resolved by consensus with a third reviewer (D.W. or N.K.Y.T.). A data extraction form was used that included: study population and setting; study design and, where applicable, follow-up duration; the number of participants at baseline and, where applicable, the number at follow-up; participant characteristics (mean age and proportion of females); the instruments used to measure pain intensity and parameters of sleep; the exposure, mediator and outcome variables examined in formal tests of mediation; the tests of mediation that were applied; and the results of these tests.

### Appraisal of Methodological Quality of Included Studies

Quality assessment of all eligible articles was undertaken in pairs (N.A. and K.K.; K.L.D. and M.B.; M.R. and D.W.), with any differences or uncertainty discussed and resolved by consensus with a third reviewer (D.W. or N.K.Y.T.). Study quality was assessed using a critical appraisal tool for experimental studies of mediation,^29^ modified for applicability to observational studies.^28^ Seven criteria assessed aspects of study design and analysis (underpinning theoretical framework, the properties of the measures used, whether the study was adequately powered, assessment of temporality (exposure-mediator and mediator-outcome), a judgement of the appropriateness of the analysis, and consideration of potentially confounding factors). In line with a number of published recommendations concerning the use of quality assessment tools,^30^–^32^ we chose not to employ a scoring system, but instead considered and narratively described each domain of interest for each study.

## RESULTS

### Study Selection

**FIGURE 2:**
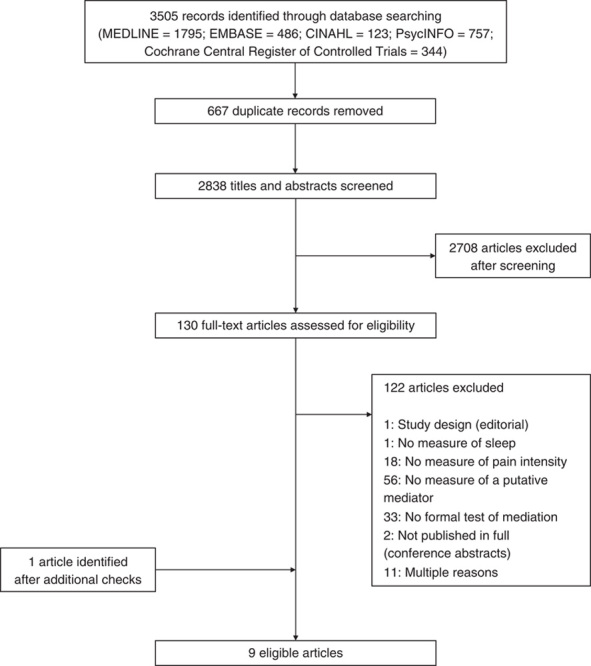
Flow of information through different phases of the systematic review.

Database searches resulted in identification of 2838 unique references. After conducting screening procedures, 130 full-text articles were retrieved, of which 8 met eligibility criteria (Fig. 2). An additional study was identified after screening reference lists of eligible articles and conducting a search of Google Scholar using key words from eligible articles. In total, therefore, 9 articles were eligible.

### Study Characteristics

Studies were published between 2008 and 2017 and conducted in the United States (6 studies), Canada, the Netherlands, and the United Kingdom (1 study each). One was experimental in design, 6 cross-sectional, and 2 longitudinal (follow-up durations of 8 weeks^33^ and 3 years^34^). The experimental study recruited healthy young adults from a US college campus. All other studies recruited clinically defined populations within which pain and sleep problems are common: children and adolescents with sickle cell disease; children, adolescents or adults with chronic pain (musculoskeletal, headache, or abdominal in origin); or adults with fibromyalgia or rheumatoid arthritis (RA). Sample sizes for mediation analyses ranged from 20 to 1415. Study characteristics are summarized in Table 1.

**TABLE 1:**
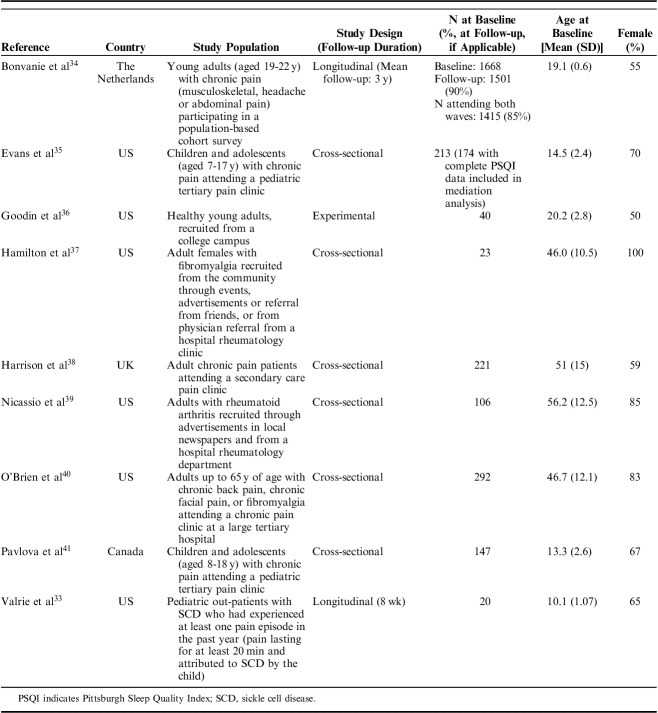
Study Characteristics

### Measures of Sleep

The tool most frequently used to measure sleep was the Pittsburgh Sleep Quality Index (PSQI). Total scores from this tool were used in 3 studies to measure sleep quality,^35^,^37^,^39^ a recognized cut-off for the tool^42^ was used to classify participants as poor (PSQI global score>5) or good sleepers (PSQI global score<5) in one study,^36^ and 2 studies created a latent variable in Structural Equation Models (SEM) using items selected from the tool to create a continuous “sleep problems”^40^ or “sleep disturbance” variable.^38^ Sleep quality in children and adolescents was measured using the revised Adolescent Sleep-Wake Scale in one study,^41^ and using a child-reported 100 mm Visual Analogue Scale (VAS) in another.^33^ In a population-based study of young adults with chronic pain, “sleep problems” were measured using the sleep scale of the Nottingham Health Profile. “Sleep problems” here were assessed with 5 questions about sleep experiences (“yes” or “no” response option): taking sleeping pills, waking up early, lying awake for most for the night, taking a long time to get to sleep, and sleeping badly at night. Each “yes” response equated to 1 point on the 0 to 5 scale.^34^

### Measures of Pain Intensity

Two studies used an 11-point Numeric Rating Scale (NRS) to measure pain intensity,^35^,^41^ and 2 used the Short-Form of the McGill Pain Questionnaire (SF-MPQ).^36^,^37^ The SF-MPQ consists of 15 items, 11 of which relate to sensory aspects of pain (eg, shooting, stabbing), and 4 to affective aspects (eg, sickening, punishing, cruel).^43^ Goodin et al^36^ used the total score from all items, whereas Hamilton et al^37^ analyzed the sensory and affective aspects as different outcomes in separate models. Single studies used an 11-point Likert scale,^34^ a 100 mm VAS,^33^ and the pain scale from the Medical Outcomes Study 36-item short-form health survey (SF-36) (0=no bodily pain to 5=very severe bodily pain).^39^ Two studies created latent pain intensity variables in SEM. This comprised the McGill Pain Questionnaire total score, VAS for average pain intensity, and usual pain intensity as measured by the Medical College of Virginia Pain Questionnaire in one study,^40^ and the 4 pain intensity items from the Brief Pain Inventory in another.^38^ For consistency, “pain intensity” is referred to throughout this review, although the term “pain severity” is used in some of the included studies. It is acknowledged that, although often used interchangeably, there are debates regarding the qualitative difference between these 2 terms. This is explored in the discussion.

### Quality Assessment

Results from an appraisal of the methodological quality of eligible studies are presented in Table 2. Two studies situated their research question and analysis within the context of existing theoretical frameworks: the sleep and pain diathesis model,^37^ and a mutual maintenance model.^41^ The sleep and pain diathesis model, an extension of the diathesis-stress model,^44^ postulates that biopsychosocial stressors, including stressful life events, may activate sleep disruption, and in predisposed individuals (those with low pain tolerance), this may then lead to maladaptive cognitions (pain helplessness in the Hamilton et al^37^ conceptualization), and subsequent experience of pain or fatigue. The mutual maintenance model tested by Pavlova et al,^41^ informed by conceptual models described by Lewin and Dahl^45^ and Valrie et al^46^ proposes that decreased sleep duration leads to increased negative affect, irritability and decreased attentional control, which then lead to increased pain perception. Sleep-related factors, including hyperarousal, play a key role in maintaining this undesirable chain of events. (This mutual maintenance model is distinct from the perpetuating link between symptoms of Post-Traumatic Stress Disorder and chronic pain as theorised by Sharp and Harvey^47^). All other studies provided a clear rationale for their mediation analyses, with previous evidence supporting cogent arguments for postulated relationships between exposure and outcome variables, exposure and mediator variables, and mediator and outcome variables.

**TABLE 2:**
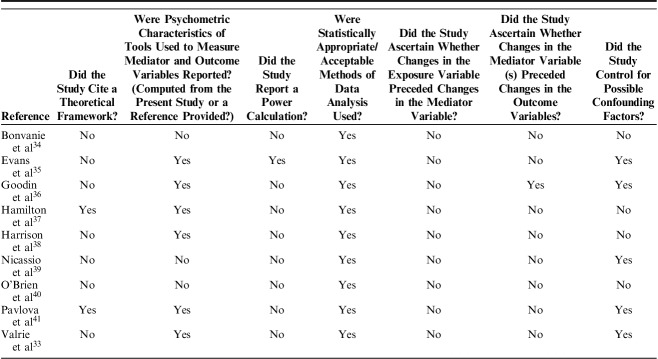
Appraisal of Methodological Quality

Six of the 9 studies reported on the psychometric properties of the tools used to measure the mediator and outcome variables, either using data collected from the sample under study or by providing a relevant reference. Of the 3 studies that did not provide such a report, one used well validated tools with established psychometric properties,^39^ one used items from established questionnaires to create latent variables in SEM,^40^ and one used a mixture of validated and nonvalidated tools.^34^ Only 1 study reported undertaking an a priori power calculation;^35^ another undertook a post hoc power analysis, providing support for an adequate sample size to test for the presence of mediation.^36^ Recognized methods to test for mediation were used in all studies.

Regarding the issue of temporality, 7 of the 9 studies were cross-sectional in design, rendering the mediation analysis exploratory and the results speculative. Of the 2 prospective studies, one collected exposure and mediator data contemporaneously at baseline and outcome data, on average, 3 years later.^34^ The other collected data using daily diaries over 8 weeks, with sleep data collected each morning and mood and pain data collected each evening.^33^ Although such intensive data collection and the application of multilevel modeling to examine within-person day-to-day variation can support a strong argument for the temporal order of change in variables, as this study collected the pain and mood variables at the same time point, it was not possible to disentangle the direction of the relationship of change in the mediator and outcome variables. Therefore, it was impossible to confidently determine the temporal ordering of variables in any of the studies included in this review (ie, to determine whether a change in the exposure led to a downstream change in the putative mediator and whether this change in the mediator was associated with a subsequent change in the outcome).

Confounding was considered in 5 of the 9 studies. One study only adjusted for the participants’ annual income,^39^ 2 adjusted for age and sex only^35^,^41^ 1 adjusted for sex, baseline level of cortisol, negative affect and duration of exposure to a cold pressor task,^36^ and another adjusted for age, sex, maternal education, sickle cell disease type, and aggregated person means for sleep and pain variables across the course of the study.^33^ Of the studies that adjusted for potential confounding, none discussed how such adjustment may have impacted on the study’s power to detect statistically significant effects.

### Mediation Analyses

**FIGURE 3:**
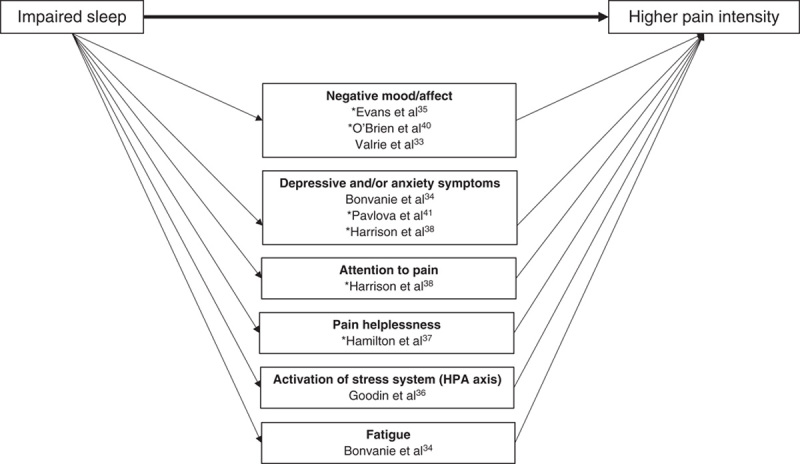
Graphical summary of factors identified as mediators on the path between sleep variables and pain intensity. *Temporal associations have not yet been well established, therefore the figure should be interpreted as hypothetical and not necessarily reflecting causality. Cross-sectional studies included in the figure are denoted with an asterisk. Statistically significant mediation has not been identified through positive affect or physical activity. However, because of methodological limitations of research undertaken to date, their role in the path from sleep to pain is far from determined. Potential confounders adjusted for: age,^33^,^35^,^41^ sex, 33,35,41 baseline level of cortisol,^36^ negative affect,^36^ duration of exposure to a cold pressor task,^36^ maternal education,^33^ Sickle Cell Disease type,^33^ aggregated person means for sleep and pain variables across the course of study.^33^

**FIGURE 4:**
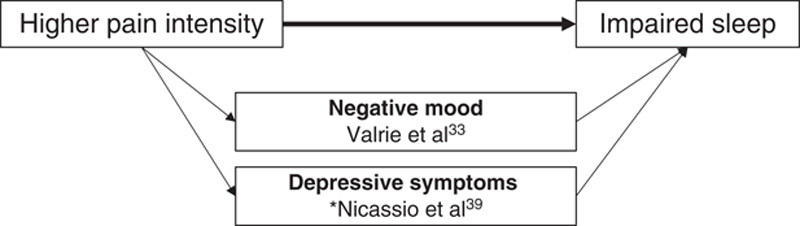
Graphical summary of factors identified as mediators on the path between pain intensity and sleep variables. *Temporal associations have not yet been well established, therefore the figure should be interpreted as hypothetical and not necessarily reflecting causality. Cross-sectional study included in the figure denoted with an asterisk. Potential confounders adjusted for: Annual income,^39^ age,^33^ sex,^33^ maternal education,^33^ Sickle Cell Disease type,^33^ aggregated person means for sleep and pain variables across the course of study.^33^

Eight of the 9 studies investigated possible mediating factors on the path from sleep to pain intensity, with 11 different pathways examined. Two articles investigated possible mediators on the path from pain intensity to sleep, with 2 pathways examined. Key findings are outlined below, detailed in Table 3, and depicted in Figures 3 and 4.

**TABLE 3:**
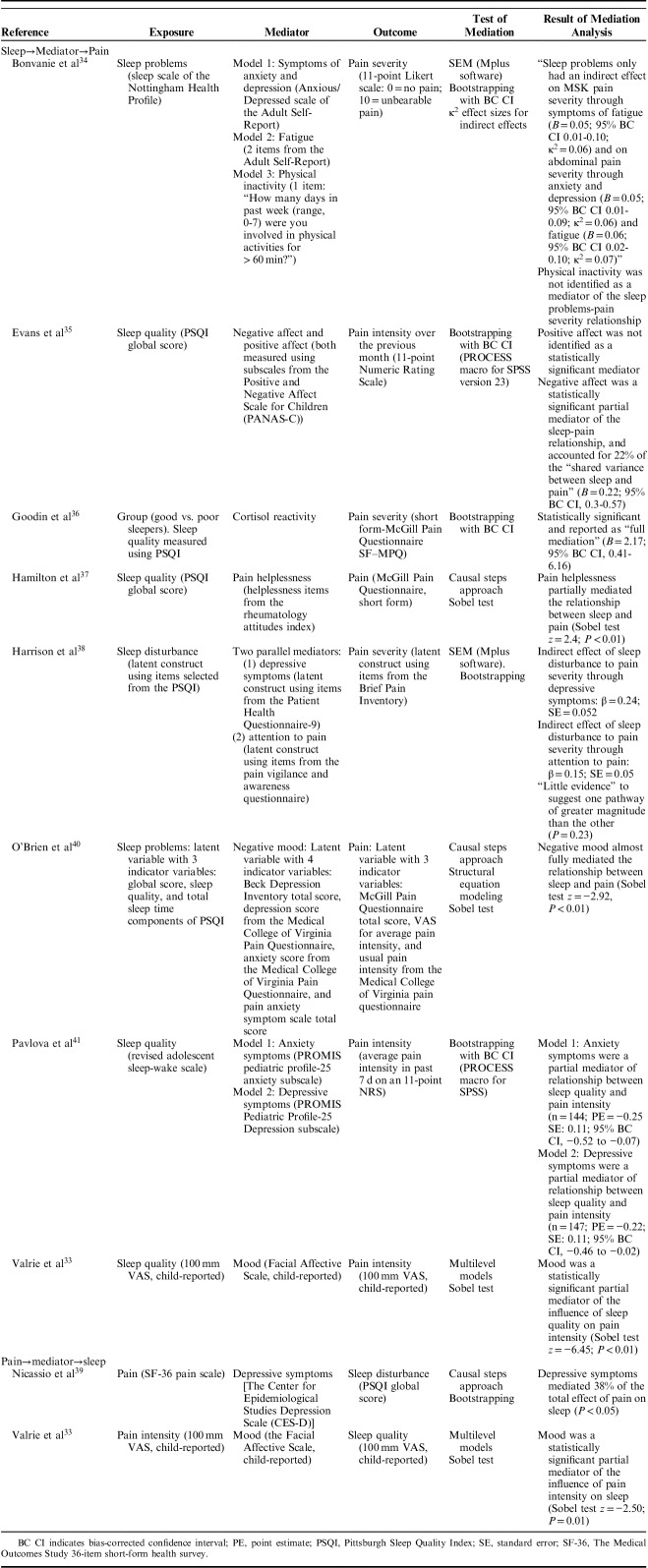
Mediation Analyses

### Mediators on the Path from Sleep to Pain

Variables investigated as possible mediators on the hypothesized path from sleep to pain could be grouped into 7 domains: affect or mood, symptoms of depression and/or anxiety, attention to pain, pain helplessness, activation of the stress system [hypothalamic pituitary adrenal (HPA) axis], fatigue, and physical activity. (Affect and mood and symptoms of depression and/or anxiety have been presented separately, reflecting the distinction previously made by Finan et al.^2^) All variables investigated within these domains were reported as carrying a statistically significant indirect effect of the exposure (sleep variable) on the outcome (pain intensity), with the exception of 2: positive affect,^35^ and physical activity.^34^

#### Affect or Mood

Evans et al^35^ hypothesized that poor sleep quality would lead to a reduction in positive affect and an increase in negative affect, and that these changes would then lead to an increase in pain intensity. In cross-sectional analysis using data collected from 174 children with chronic pain attending a tertiary pain clinic, guided by Baron and Kenny^21^ causal steps criteria, they determined that positive affect was not a mediator of the sleep-pain relationship as it was not significantly associated with pain intensity. However, application of Baron and Kenny criteria provided support for a mediating role for negative affect and this variable was then further analyzed using the Preacher and Hayes^48^ bootstrapping procedure. Results identified negative affect as responsible for an estimated 22% of the total effect of poor sleep quality on pain intensity (analysis adjusted for child age and sex).

In unadjusted analysis using data from 292 adults with chronic pain, O’Brien et al^40^ used structural equation modeling (SEM) to examine the hypothesis that negative mood is a mediator of the “sleep problems”-pain relationship. The cross-sectional relationship between “sleep problems” and pain was almost completely eliminated after negative mood was entered into their model (path coefficient reduced from −0.51 to −0.02), and a case for mediation was supported by a statistically significant Sobel test. This led the authors to argue that negative mood almost fully explained the “sleep problems”-pain relationship.

Valrie et al^33^ used data collected from daily diaries over 8 weeks to determine whether mood mediated the sleep quality-pain intensity relationship in children with sickle cell disease. Using results obtained from multilevel modeling and a statistically significant Sobel test, they reported that poor sleep quality predicted higher pain intensity the following day, and that this relationship was mediated by negative mood. This analysis was adjusted for age, gender, level of maternal education, sickle cell disease type, and aggregated person means for sleep and pain variables.

#### Symptoms of Depression and/or Anxiety

Bonvanie et al^34^ collected baseline responses to the sleep scale of the Nottingham Health Profile from a population-based cohort of young adults with chronic pain (the exposure variable in their analysis) and the anxious/depressed scale of the adult self-report (the putative mediator, also collected at baseline). Three outcomes were examined in 3 separate models using data collected, on average, 3 years later: severity of musculoskeletal pain, headache/migraine, and abdominal pain (0=no pain, 10=unbearable pain). Using unadjusted bias-corrected bootstrapping, symptoms of anxiety and depression were reported as a statistically significant mediator of the relationship between “sleep problems” and abdominal pain severity (*B*=0.05; 95% CI, 0.01-0.09, κ^2^=0.06), but not musculoskeletal or headache/migraine pain severity.

In a study of chronic pain patients attending a UK secondary care clinic, Harrison et al^38^ investigated the potentially intermediary role of depressive symptoms and attention to pain as parallel mediators between sleep disturbance (a latent construct created from items selected from the PSQI) and pain intensity (a latent construct using items from the BPI). Cross-sectional, unadjusted analysis using SEM identified both putative mediators as carrying a statistically significant effect of the exposure on the outcome (indirect effects: depressive symptoms β=0.24, SE=0.05; attention to pain β=0.15, SE=0.05), with no evidence to suggest that either carried substantially more of the effect.

In 2 separate cross-sectional analyses adjusted for age and sex, Pavlova et al^41^ identified both anxiety and depressive symptoms as partial mediators of the sleep quality-pain intensity relationship in children and adolescents with chronic pain attending a tertiary pain clinic. In separate models, 45% of the total effect of sleep quality on pain intensity was explained by a pathway through anxiety symptoms, and 41% of the effect was explained through depressive symptoms.

#### Attention to Pain

As described above, when analyzed in parallel to depressive symptoms, Harrison et al^38^ identified attention to pain as a statistically significant partial mediator of the relationship between sleep disturbance and pain intensity (indirect effect β=0.15, SE=0.05, unadjusted, cross-sectional analysis).

#### Pain Helplessness

Testing part of the Sleep and Pain Diathesis model, Hamilton et al^37^ undertook cross-sectional analysis of data collected from 23 females with fibromyalgia. They hypothesized that more disturbed sleep would be associated with greater dysfunctional cognitions about pain, specifically a perception of pain helplessness, and that this would be associated with higher pain intensity. Using responses to the sensory dimension of pain subscale of the SF-MPQ as the outcome in unadjusted analyses, a statistically significant association was identified with sleep disruption (measured using the PSQI). This association attenuated after adjustment for pain helplessness (measured using the Rheumatology Attitudes Index), from β=0.10 (SE=0.04) to β=0.04 (SE=0.04), and a statistically significant result from a Sobel test (*P*<0.01) was used to support a case for mediation.

#### Activation of the Stress System (HPA Axis)

In the only study to investigate a biological mechanism, Goodin et al^36^ applied mediation analysis to examine the predictive capacity of sleep quality on neuroendocrine stress reactivity (specifically cortisol reactivity) and the subsequent pain reporting in response to physical stress [a cold pressor task (CPT)]. Forty healthy young adults were categorized as poor or good sleepers using an established cut-off score on the PSQI (>5=poor, ≤5=good^42^), and salivary cortisol samples were collected before initiation of the CPT and 15, 20, 25, 30, and 40 minutes later. After completing the CPT, participants reported their pain intensity using the SF-MPQ. In analysis that adjusted for sex, baseline level of salivary cortisol, negative affect, and duration of exposure to the CPT, poorer sleepers were found to have an exaggerated cortisol response to the CPT, and this was associated with significantly higher pain severity scores.

#### Fatigue

In addition to their examination of symptoms of anxiety and depression as putative mediators of the sleep problems-pain intensity relationship, Bonvanie et al^34^ also used baseline responses to the sleep scale of the Nottingham Health Profile (exposure) and the mean of 2 fatigue items from the adult self-report (the putative mediator, data also collected at baseline). The same 3 outcomes were examined in 3 separate analyses using data collected, on average, 3 years later (severity of musculoskeletal, headache/migraine, and abdominal pain). Using unadjusted bias-corrected bootstrapping, fatigue was reported as a statistically significant mediator of the relationship between sleep problems and abdominal pain intensity (*B*=0.06; 95% CI, 0.02-0.10; κ^2^=0.07), and sleep problems and musculoskeletal pain severity (*B*=0.05; 95% CI 0.01- 0.10; κ^2^=0.06), but not between sleep problems and headache/migraine pain severity.

#### Physical Activity

Bonvanie et al^34^ conducted a further, similar analysis with the same dataset, this time using a crude, single item about physical activity collected at baseline as the putative mediator (self-report of number of days in the past week during which the participant undertook >60 min of physical activity that raised their breathing or heart rate; range, 0 to 7). Physical activity was not identified as a mediator of the sleep problem-pain severity relationship for any of the 3 outcomes (severity of musculoskeletal, headache/migraine, or abdominal pain).

### Mediators on the Path from Pain to Sleep

Two variables were investigated as possible mediators of the pain-sleep relationship: depressive symptoms and mood.

#### Depressive Symptoms

Depressive symptoms, measured using the Center for Epidemiological Studies Depression Scale, was reported by Nicassio et al^39^ as a partial “mediator” of the cross-sectional relationship between pain intensity (SF-36 pain scale) and sleep disturbance (global PSQI score) in adults with RA. In analysis that adjusted for the participants’ level of annual income, an estimated 38% of the total effect of pain intensity on sleep disturbance was explained through this indirect pathway.

#### Mood

Mood, reported by children with sickle cell disease using the facial affective scale, was identified as a statistically significant partial mediator of the longitudinal relationship between pain intensity and sleep quality, both measured using a child-reported 100 mm VAS, in an analysis that adjusted for child age, sex, level of maternal education, sickle cell disease type and aggregated person means for the sleep and pain variables.^33^ The results suggest that higher levels of sickle cell disease-related pain are associated with lower mood, which has a downstream effect on sleep quality.

## DISCUSSION

The aim of this systematic review was to identify, synthesize, and critically appraise studies that have investigated potentially mediating variables on the path between sleep variables and pain intensity (or the reciprocal relationship) using a formal test of mediation. Nine studies were identified, with a variety of putative mediators investigated in different populations using a range of statistical approaches. Eleven analyses investigated mediators on the path from sleep to pain (mood, pain helplessness, symptoms of anxiety and depression, negative and positive affect, attention to pain, fatigue, physical activity, and cortisol reactivity), and 2 investigated potential mediator variables on the path from pain to sleep (depressive symptoms and mood). Of these, only positive affect and physical activity were not identified as mediators. However, these findings are not conclusive. Methodological limitations, including the measurement instruments used, timing of data collection, and lack of adjustment for potential confounders render both the statistically significant and null findings far from definitive.

The majority of pathways examined in studies included in this review were simple, single, or parallel mediator models. A trend over time can be observed with increased application of SEM. However, the potential benefits of SEM in allowing measurement error to be accounted for, and shared variance within a single instrument when used repeatedly to measure a given construct, have not, to date, been fully exploited in this field. The complexity of expanded causal relationships that link sleep variables and pain intensity demand more intricate theoretical models, including those that acknowledge subgroup heterogeneity and moderated pathways that take the influence of affective and cognitive factors on pain perception into consideration.^49^–^51^ Before embarking on more complex modeling strategies, however, the current state of the evidence requires critical evaluation.

In the narrative description of the results of each study we have provided information about important aspects of study design and analysis to facilitate balanced consideration of the strength of evidence for mediation. Overall, quality assessment of studies included in the review revealed methodological limitations of both design and analysis. This echoes comparable findings from systematic reviews of studies of mediation from across the applied health sciences literature^28^,^52^–^55^ and their critique.^56^ Given the potentially nontrivial effects of these limitations on the results and interpretation of studies of mediation, recommendations for optimal conduct and analysis is required. It should be noted, however, that quality assessment criteria—which can be used to guide research design—have only been formally outlined relatively recently for studies of mediation. The fact that over time studies have been attending to a number of key quality domains is encouraging and greater uptake of the recommendations will improve on-going research practice and reporting.

### Recommendation 1: Development of a Unified Conceptual Model

A lack of a unified conceptual framework that links pain, sleep, physiology, psychosocial factors, and behavior could be argued to hamper integrated progress in this field. Such a framework may be useful in informing the construction of a priori hypothesized directed acyclic graphs (DAGs). DAGs are graphical representations of hypothesized causal pathways, informed by theory and existing empirical evidence. Their construction facilitates consideration of potentially confounding variables at all stages of the hypothesized causal path.^22^,^57^,^58^ A model specific to a pediatric persistent pain population has been outlined, a result of a systematic review of the relationship between pain and sleep in this population,^46^ highlighting the potentially mediating role of mood. A conceptual framework specific to osteoarthritis, described by Smith et al,^59^ has also implicated augmented central pain processing and basal inflammation. To build on these models, a conceptual framework that includes the expected period within which changes in variables are hypothesized to occur would be extremely helpful to inform future research. This could direct the frequency and timing of data collection procedures. A unified conceptual framework may be particularly important as it is likely that researchers will continue to examine more complex models of the sleep-pain relationship, with multiple mediators and moderated pathways.

### Recommendation 2: The Use of (micro) Longitudinal Study Designs to Investigate Causal Mediation

The only way to determine whether the hypothesized temporal order of changes is empirically upheld is through the use of prospectively collected data. The majority of the studies in the current review were cross-sectional in design and their arguments for causality therefore remain speculative. However, prospectively collecting data does not in itself allow the temporal order of changes to be determined, and even in cases where study design and analysis make use of data collected at multiple time points, the importance of being able to disentangle the order of changes requires greater consideration, both from a design and analysis point of view. For example, in the prospective study by Valrie et al,^33^ negative mood and pain intensity were both measured in the evening, therefore weakening a case for causality. Also open to questions of temporal ambiguity is the study by Goodin et al^36^ in which CPT-induced cortisol reactivity was hypothesized as a mediator of the sleep quality-acute pain intensity relationship. Given the testing schedule (collection of salivary cortisol prior to and after CPT, and pain report after CPT), cortisol reactivity could have been conceptualized and examined as the outcome, not the mediator. Indeed, it is likely that an exaggerated pain response in poor sleepers produces an amplified activation of the HPA axis response to stress, particularly cortisol reactivity.

The studies that provide the richest data apply microlongitudinal designs using ecological momentary assessment, thereby allowing within-person variations in a range of symptoms to be examined, as well as adjustment for person-level variables. Combined with multilevel and latent growth curve modeling approaches,^60^ these methods hold much promise to address questions of causal mediation.

### Recommendation 3: Optimize Measurement

Temporality must also be borne in mind when selecting the tools with which to measure the variables under examination. The PSQI was the most frequently used tool to measure sleep. This tool asks about sleep experiences over the past month. In addition to this collapsing of time, the self-report nature of this instrument renders its output subject to recall bias; the crystallized 1-month data is likely to better represent proximal rather than distal sleep experiences. The increasing availability and use of clinical grade actigraphy in research and, more generally, wearable devices that passively monitor sleep, provide an alternative, possibly additional, objective measure that may better capture momentary changes in sleep variables over time. Future studies would benefit from collecting objective data on parameters of sleep, in addition to participant-reported variables.

In contrast, pain, as a subjective, multifaceted experience, cannot be passively and objectively measured. Although dolorimetry may be used to quantify pain intensity, on-going passive measurement is not possible. Retrieving frequent longitudinal data on pain intensity using paper or electronic diaries increases participant burden, may introduce reactivity, and has the potential to reduce study generalizability as a consequence of possible selection bias introduced through the demands of participation. Furthermore, although digital technologies (eg, smartphone applications) are increasingly used to intensively collect data on self-reported pain intensity [ie, (multiple measurements in a given day)], standardization of their application has been argued as requiring attention.^61^ These complex and challenging issues demand on-going attention; certainly, if the results of studies of mediation are to be robust to scrutiny, it is essential that the tools, frequency and timing of measurement of variables is adequate for analysis.

Furthermore, it is essential that specific facets of sleep and pain are studied, ideally capturing the multifaceted nature of these experiences. Studies in the current review investigate a number of different parameters of sleep, not all of which are comparable. Although more alike, the inclusion of studies that investigated either pain intensity or pain severity potentially obfuscates the qualitative nature of pain included in the severity measure. Jensen et al^62^ have demonstrated that pain severity ratings (ie, mild, moderate, or severe) are likely to reflect not just the intensity of the pain but also its interference, catastrophizing aspects, and pain-related beliefs. Given the continuous measures used to measure pain in studies included in this review, for consistency we refer to pain intensity throughout. It should be borne in mind, however, that, despite our inclusion criterion, it is unrealistic to assume that we have captured data on pain intensity only.

Using appropriate, valid and reliable tools to measure putative mediator variables is also imperative; crude measurement tools reduce the likelihood of detecting changes. An example of this is the use of a single self-reported item to capture information about physical activity in the study by Bonvanie et al,^34^ which found physical activity not to be a mediator. They investigated the potentially mediating role of 3 candidate variables in 3 separate analyses: anxiety and depression, fatigue, and physical activity, variables with the potential for statistical overlap. On request for further details, the authors provided correlation coefficients which exhibited a significant association between baseline levels of anxiety/depression and fatigue in the expected direction (higher levels of anxiety/depression associated with higher levels of fatigue). There were no significant associations between anxiety/depression and physical activity, or fatigue and physical activity. This surprising finding is likely also attributable to the crude measure of physical activity used.

### Recommendation 4: Improved Analysis

A general shift over time can be observed from application of the traditional causal steps approach to path analytic approaches and the use of structural equation and multilevel models. However, the more sophisticated methods of analysis often require larger sample sizes, a feature rarely discussed across studies. Indeed, over half of the studies (5/9) recruited <150 participants, previously cited as the minimum number to detect mediating effects and avoid type II error.^28^,^63^ Although such rule of thumb guidance is questionable (an appropriate sample size for an adequately powered study of mediation depends upon a number of variables, not least the parsimony or complexity of the model and the number of adjustments made), it is undeniable that most studies to date have recruited relatively small numbers of participants. The fact that these studies almost always identified statistically significant mediating effects even though they may have been underpowered to do so indicates the possibility of overestimation—a particular concern in studies of mediation when confounding has not been adequately adjusted for. The types of factors that are examined in this field of research are complex, multifactorial constructs that may comprise biological, psychological, social, emotional, and behavioral aspects. Confounding of relationships between these factors by other variables is highly likely. With regard to estimates of precision, where Preacher and Hayes^48^ procedures were applied, bias-corrected confidence intervals were exclusively reported. However, percentile-corrected (PC) confidence intervals have been cogently argued as preferable.^64^ It is therefore recommended that future bootstrapping approaches report PC estimates. Given the exploratory nature of studies of mediation in the sleep-pain field to date, more detailed causal mediation analysis procedures are yet to be applied. However, as research in this area continues, there is an opportunity to apply contemporary developments in causal inference methods. Specific practices that would benefit from thoughtful implementation include: more thorough consideration of confounding on the putative causal pathway (possible confounders of the exposure-outcome, exposure-mediator, and mediator-outcome relationships); investigations of interactions between exposure and mediator variables (important in the analysis of data collected in the context of RCTs, notably yet to be exploited in the sleep-pain field);^65^ taking into account multiple possible mediators on the causal path;^66^ and the conduct of sensitivity analyses to investigate the impact of residual confounding.^22^,^23^

### Recommendation 5: Improved Reporting

Future research would benefit from more transparent reporting of the statistical methods used to assess mediation. Journal word limits can be a barrier to comprehensive reporting, however, a study asking a closely related question (whether poor quality sleep leads to increased pain intensity, and if this is then associated with onset of temporomandibular disorder)^67^ provides an extremely helpful and comprehensive explanation using supplementary files. In addition, consistent and thorough reporting of the regression coefficients of all pathways in the mediation analyses (exposure to mediator, mediator to outcome, direct, indirect, and total effects) and their standard errors would improve transparency and support future meta-analyses.

### Gaps in the Evidence Base

In their review of the association between sleep and pain, Finan et al^2^ outline 3 major biobehavioral mechanisms that may link sleep and pain, namely dopaminergic signaling, opioiderigic signaling, and negative and positive affect. Of these, only the latter 2 have begun to be directly investigated in studies that have used formal tests of mediation (described in this review). In a more recent overview, Nijs et al^16^ draw attention to the potentially influential role of serotoninergic pathways and, drawing on data from experimental (largely preclinical) research, the potential importance of relationships between sleep deprivation (along with severe and/or chronic stress), abnormal glial activation, and (neuro)inflammation,^15^ altered levels of circulating proinflammatory cytokines (eg, TNFα, IL-6)^68^,^69^ and the onset or persistence of hyperalgesia and central sensitization.^70^,^71^ The complexity and almost certain interconnectedness of the causal web is alluded to by the fact that neuroinflammation is a feature of major depressive disorder, and that it may be mitigated by physical activity.^72^ Applying advances in mediation analysis to unravel these connections in the future holds much promise.

Notably lacking from the current evidence base are studies focused on mediators of the sleep-pain relationship in people living with cancer. However, a number of studies focused on this clinical population have investigated sleep variables or pain intensity as mediators themselves on pathways between pain and fatigue,^73^ quality of life and sleep,^74^ sleep and fatigue, mood and pain,^75^ pain intensity and function,^76^ and rest-activity rhythms and sleep quality.^77^ All of these studies are cross-sectional in design, a common limitation of the investigations of mediators of the sleep-pain relationship undertaken to date. Any future studies of mediators of the sleep-pain relationship in people living with cancer are encouraged to undertake prospective data collection and consider our recommendations.

Also lacking are studies into the potentially influential role of diet in the sleep-pain relationship. In previous research associations have been demonstrated between dietary factors and sleep,^78^–^80^ and between diet and pain intensity.^81^ Dietary constituents have also been shown to modulate mood^82^ and stress,^83^ with hypothesized mechanisms implicating the neuroendocrine system.^84^ To date this remains an unexplored area with regard to mediation analysis.

This review itself is subject to limitations. Synthesis of the evidence was restricted to narrative description because of the variety of mediators and the way they were analyzed across different studies. This prevented comparison and balanced consideration of key pathways that may be exploited by management approaches that aim to improve pain and/or sleep. Indeed, the range of statistical approaches used prevented comparison of the magnitude of effects, and evidence to support arguments for mediation were often reduced to findings from tests of statistical significance. It is important to be able to compare the magnitude of mediating effects across studies to identify the most influential pathways and therefore the most viable targets for interventions that aim to relieve burdensome symptoms. Complete reporting of all pathways in proposed mediation models would allow for such comprehensive comparison and this practice is recommended in future reporting. Regardless of the inability to pool estimates from the currently available evidence, given the limitations of the methods, any kind of quantitative synthesis was deemed inappropriate.

The review was also limited by restricting its eligibility criteria to models that include a measure of pain intensity specifically. This meant that studies focused on, for instance, pain interference rather than intensity were excluded. This strategy was adopted to focus the search and comprehensively appraise studies that have concentrated on 1 facet of pain. Also, the relationship between pain intensity and pain interference or disability is itself potentially mediated by psychosocial variables and has been the subject of previous systematic reviews of studies of mediation.^28^,^85^ Even after putting in place such a restriction, the qualitative differentiation of pain intensity, pain severity, and the sensory and affective aspects of pain meant that there was some heterogeneity in the pain variables in included studies. Publication bias is also a possibility, and it should be noted that we did not undertake a formal examination to assess its possible extent. The vast majority of studies provided evidence to support a case for the mediating pathways that they investigated. It is likely that in cases where post hoc mediation analyses have been undertaken, only those with statistically significant results have been reported. It is unlikely, however, that unpublished studies, if they exist, have superior methods to those in the current review; high quality mediation analyses applied to this clinically important question are needed.

We must acknowledge that in restricting our search to studies that conducted a formal test of mediation other types of evidence that could provide important insights into the mechanisms underlying the sleep-pain relationship were not included. The process of developing a case for causal inference can itself be viewed as a temporal process, each stage lying on a continuum, with theoretically informed cross-sectional analyses at one pole and randomised controlled trials with embedded mechanistic studies analyzed using mediation analysis at the other. A notable investigation lying on this continuum is a randomized study by Haack et al^69^ that identified a relationship between sleep restriction, elevated inflammatory markers (IL-6), and increased pain ratings. However, in focusing on studies that formally tested for mediation, our review allowed the strength of causal arguments using this approach to be rigorously assessed, and the use of these methods in the sleep-pain field to date established, informing recommendations for future practice.

In conclusion, there is a relatively small body of research that has formally tested indirect pathways between sleep variables and pain intensity. On the basis of the reviewed evidence, we can speculate that psychological and physiological components of emotional experience and attentional processes are likely mediators of the sleep-pain relationship. Although the hypothesized mediating effect of physical activity has not been supported by current evidence, its role can not be ruled out. Because of methodological limitations inherent to cross-sectional studies, measurement imprecision, and potentially overlapping constructs, as well as greater scope for theory-based investigations, there is a need for evidence underpinned by optimally designed and conducted studies of mediation. This research area holds much promise for informing the development of multimodal pain management programmes and, through the future use of data collected within the context of RCTs, investigating *how* sleep improvement interventions may affect pain intensity over time. However, at present the design and analysis of studies of mediation requires greater attention and replication of findings after methodological improvements are consolidated.

## Supplementary Material

SUPPLEMENTARY MATERIAL

Supplemental Digital Content is available for this article. Direct URL citations appear in the printed text and are provided in the HTML and PDF versions of this article on the journal's website, www.clinicalpain.com.
