# Supplementary material for: Sleep and Pain: A Systematic Review of Studies of Mediation
Source: Clin J Pain. 2019 Mar 1;35(6):544–58. doi: 10.1097/AJP.0000000000000697 (PMC6504189; doi:10.1097/AJP.0000000000000697)
Supplement: SUPPLEMENTARY MATERIAL [file ajp-35-544-s001.docx]

**Supplement 1. Database search strategy**

**OVID databases**

(mediat* OR indirect OR "structural equation modeling" OR "structural equation modelling" OR "path" OR "Baron and Kenny" OR MacKinnon OR "product of coefficient" OR "difference in coefficient" OR "process of change" OR sobel* OR "causal pathway" OR intermediate OR "indirect effect" OR "process variable" OR "treatment ADJ2 effect" OR "process ADJ2 evaluation" OR mechanism OR SEM OR bootstrap* or caus*).tw

**AND**

pain*.tw

**AND**

(sleep* or insomni*).tw

**RESTRICT** to ‘Humans’

***CINHAL database***

(mediat* OR indirect OR "structural equation modeling" OR "structural equation modelling" OR "path" OR "Baron and Kenny" OR MacKinnon OR "product of coefficient" OR "difference in coefficient" OR "process of change" OR sobel* OR "causal pathway" OR intermediate OR "indirect effect" OR "process variable" OR "treatment ADJ2 effect" OR "process ADJ2 evaluation" OR mechanism OR SEM OR bootstrap* or caus*)

**AND**

pain*

**AND**

(sleep* or insomni*)

**RESTRICT** to ‘Humans’
